# Supplementary material for: EnzML: multi-label prediction of enzyme classes using InterPro signatures
Source: BMC Bioinformatics. 2012 Apr 25;13:61. doi: 10.1186/1471-2105-13-61 (PMC3483700; doi:10.1186/1471-2105-13-61)
Supplement: Addtional file 5 — The Java code to format the data files, evaluate and predict. The file enzml_java_code.tar.gz contains the Java code used to format database data to ARFF and XML formats, to execute cross and train-test (jackknife) evaluations and to record evaluation results to database. More information is included in the readme.txt file and the Javadoc files. The code can be used with a MySQL database. To use a different database software, other JDBC drivers might be required. [file 1471-2105-13-61-S5.gz › java_code/utils/doc/uk/ac/ed/inf/utils/database/DbConn.html]

DbConn


---


|  |  |  |
| --- | --- | --- |
| **Overview** \| **Package** \| **Class** \| **Use** \| **Tree** \| **Deprecated** \| **Index** \| **Help** | |  |
| PREV CLASS   **NEXT CLASS** | **FRAMES**    **NO FRAMES**     **All Classes** |  |
| SUMMARY: NESTED \| FIELD \| CONSTR \| METHOD | DETAIL: FIELD \| CONSTR \| METHOD |  |


---


## uk.ac.ed.inf.utils.database Class DbConn

```
java.lang.Object
  uk.ac.ed.inf.utils.database.DbConn
```

---

``` public class DbConn extends java.lang.Object ```

A DbConnectionProperties object contains the properties needed to connect to
a database through JDBC. The properties are read from the default properties
file or from a given file.
DbConnectionProperties also generates a new connection and statement.
The Java properties file is a text file in the form:
# Database type databaseType=mysql # JDBC driver (comma-separated list)
jdbcDriver=org.gjt.mm.mysql.Driver # Connection parameters host=localhost
port=3306 database=mydb user=myuser pswd=secretpassword

**Version:**
:   18-Apr-2007

**Author:**
:   ldeferra

---

| **Field Summary** | |
| --- | --- |
| `static java.lang.String` | `DATABASE_NAME_PROP` |
| `static java.lang.String` | `DATABASE_TYPE_PROP` |
| `static java.lang.String` | `HOST_PROP` |
| `static java.lang.String` | `JDBC_DRIVER_PROP` |
| `static java.lang.String` | `PASSWORD_PROP` |
| `static java.lang.String` | `PORT_PROP` |
| `static java.lang.String` | `USER_PROP` |


**Constructor Summary**

`DbConn(java.lang.String propertiesFilePath)`
  
          Constructor: takes connection parameters from the specified properties
file


| **Method Summary** | |
| --- | --- |
| `void` | `closeConnection()`             Close the database connection |
| `boolean` | `connectionIsValid()` |
| `static boolean` | `connectionIsValid(java.sql.Connection connection)`             Checks that the connection is not null |
| `java.sql.Connection` | `createConnection()`             Creates a new connection to the database |
| `java.sql.Connection` | `getConnection()` |
| `java.lang.String` | `getConnectionParametersString()`             Get the connection log (database driver + url) |
| `java.lang.String` | `getDatabaseName()`             Gets the database the connection is connected to |
| `java.lang.String` | `getDatabaseType()` |
| `java.lang.String` | `getDriverName()` |
| `java.lang.String` | `getHost()` |
| `DbConn` | `getOpenConnection()` |
| `java.lang.String` | `getPassword()` |
| `java.lang.String` | `getPort()` |
| `java.sql.Statement` | `getStatement()`             Gets a statement from a new connection |
| `java.lang.String` | `getUrl()` |
| `java.lang.String` | `getUser()` |
| `void` | `readConnectionProperties(java.lang.String propertiesFilePath)`             Reads the connection properties from the java properties file specified in the path |

**Methods inherited from class java.lang.Object**

`equals, getClass, hashCode, notify, notifyAll, toString, wait, wait, wait`

**Field Detail**

### DATABASE\_NAME\_PROP

```
public static final java.lang.String DATABASE_NAME_PROP
```

**See Also:**: Constant Field Values

---


### DATABASE\_TYPE\_PROP

```
public static final java.lang.String DATABASE_TYPE_PROP
```

**See Also:**: Constant Field Values

---


### HOST\_PROP

```
public static final java.lang.String HOST_PROP
```

**See Also:**: Constant Field Values

---


### JDBC\_DRIVER\_PROP

```
public static final java.lang.String JDBC_DRIVER_PROP
```

**See Also:**: Constant Field Values

---


### PASSWORD\_PROP

```
public static final java.lang.String PASSWORD_PROP
```

**See Also:**: Constant Field Values

---


### PORT\_PROP

```
public static final java.lang.String PORT_PROP
```

**See Also:**: Constant Field Values

---


### USER\_PROP

```
public static final java.lang.String USER_PROP
```

**See Also:**: Constant Field Values


**Constructor Detail**

### DbConn

```
public DbConn(java.lang.String propertiesFilePath)
```

:   Constructor: takes connection parameters from the specified properties
    file


**Method Detail**

### closeConnection

```
public void closeConnection()
```

:   Close the database connection

---


### connectionIsValid

```
public boolean connectionIsValid()
```

---


### createConnection

```
public java.sql.Connection createConnection()
```

:   Creates a new connection to the database

    :   **Returns:**: a connection to the database

---


### getConnection

```
public java.sql.Connection getConnection()
```

---


### getConnectionParametersString

```
public java.lang.String getConnectionParametersString()
```

:   Get the connection log (database driver + url)

    :   **Returns:**: the connection log (database driver + url)

---


### getDatabaseName

```
public java.lang.String getDatabaseName()
```

:   Gets the database the connection is connected to

    :   **Returns:**: the database name

---


### getDatabaseType

```
public java.lang.String getDatabaseType()
```

---


### getDriverName

```
public java.lang.String getDriverName()
```

---


### getHost

```
public java.lang.String getHost()
```

---


### getOpenConnection

```
public DbConn getOpenConnection()
```

---


### getPassword

```
public java.lang.String getPassword()
```

---


### getPort

```
public java.lang.String getPort()
```

---


### getStatement

```
public java.sql.Statement getStatement()
```

:   Gets a statement from a new connection

    :   **Returns:**: a database statement

---


### getUrl

```
public java.lang.String getUrl()
```

---


### getUser

```
public java.lang.String getUser()
```

---


### readConnectionProperties

```
public void readConnectionProperties(java.lang.String propertiesFilePath)
```

:   Reads the connection properties from the java properties file specified
    in the path

    :   **Parameters:**: `propertiesFilePath` - the path to the java properties file containing the connection parameters

---


### connectionIsValid

```
public static boolean connectionIsValid(java.sql.Connection connection)
```

:   Checks that the connection is not null

    :   **Parameters:**: `connection` - **Returns:**: true if the connection is not null or has not been closed


---


|  |  |  |
| --- | --- | --- |
| **Overview** \| **Package** \| **Class** \| **Use** \| **Tree** \| **Deprecated** \| **Index** \| **Help** | |  |
| PREV CLASS   **NEXT CLASS** | **FRAMES**    **NO FRAMES**     **All Classes** |  |
| SUMMARY: NESTED \| FIELD \| CONSTR \| METHOD | DETAIL: FIELD \| CONSTR \| METHOD |  |


---
